# Droplet Digital PCR: A New Molecular Method to Detect G1105S/V Mutations in *Plasmopara viticola CesA3* Gene

**DOI:** 10.3390/biology13110919

**Published:** 2024-11-13

**Authors:** Helene Sánchez-Zelaia, Irene Maja Nanni, Ivano Oggiano, Mónica Hernández, Ana María Díez-Navajas, Marina Collina

**Affiliations:** 1 Department of Plant Production and Protection, NEIKER-Basque Institute of Agricultural Research and Development, Basque Research and Technology Alliance (BRTA), Campus Agroalimentario de Arkaute, 01192 Arkaute, Spain; hsanchez@neiker.eus (H.S.-Z.); mhernandez@neiker.eus (M.H.); adiez@neiker.eus (A.M.D.-N.); 2 Department of Agricultural and Food Sciences (DISTAL), University of Bologna, 40127 Bologna, Italy; irenemaja.nanni2@unibo.it (I.M.N.); ivano.oggiano2@unibo.it (I.O.)

**Keywords:** Carboxylic Acid Amides, ddPCR, *Plasmopara viticola*, fungicide resistance, *CesA3*

## Abstract

*Plasmopara viticola* is the causal agent of Grapevine Downy Mildew (GDM), which is a devastating disease of grapevines in humid temperate regions. The most employed method for protecting grapevines against GDM is the application of chemical fungicides. In Spain, Carboxylic Acid Amides (CAAs) are a fungicide group currently utilized in GDM control. In *P. viticola*, resistance to CAAs is conferred by G1105S and G1105V mutations in the *CesA3* gene. Droplet digital polymerase chain reaction (ddPCR) is an innovative technique that combines PCR and droplet microfluidics to disperse the sample into thousands of water-in-oil droplets in which an amplification reaction is individually performed. In this study, we set up a ddPCR protocol to quantify S1105 and V1105 mutations conferring resistance to CAAs in *P. viticola*. The optimal PCR conditions were established, and the sensitivity and precision of the protocol were assessed. Four *P. viticola* populations coming from commercial vineyards in northern Spain were analyzed, and different allele frequencies were found in the analyzed samples corresponding to the different fungicide management strategies, ranging from 7.72% to 100%. Knowing the level of mutated alleles allows for designing resistance management strategies suited for each location. This suggests that similar ddPCR assays could be developed for studying mutations implicated in fungicide resistance in other fungicide groups and plant pathogens.

## Simple Summary

Fungicide resistance is the natural and inheritable adaptation of pathogens to survive treatment with a phytosanitary product that would normally provide effective control. *Plasmopara viticola*, the causal agent of Grapevine Downy Mildew (GDM), is an important pathogen in vineyards, in which resistance to Carboxylic Acid Amide (CAA) fungicides has been observed and reported. Behind this resistance, there are two single-point substitutions of the cellulose synthase gene: G1105S and G1105V. In this article, we developed a droplet digital polymerase chain reaction (ddPCR) protocol for the quantification of the mutations conferring the resistance. The ddPCR protocol precisely determined allele frequencies in four fields where *P. viticola* bulk samples were collected.

## 1. Introduction

*Plasmopara viticola* (Berk. & M.A. Curtis) Berl. & de Toni, the causal agent of Grapevine Downy Mildew (GDM), is responsible for severe disease epidemics causing great yield losses. The use of chemical fungicides is an effective strategy to protect grapevines against *P. viticola* infection, but the continuous use of fungicides with the same modes of action can lead to the development of resistant strains, and problems with disease control can occur when resistant individuals become predominant over sensitive individuals [1]. Moreover, according to FRAC (Fungicide Resistance Action Committee) [2], *P. viticola* is a high-risk pathogen for fungicide resistance development.

Carboxylic Acid Amide (CAA) fungicides have been employed in Spanish vineyards for many years since the use of dimethomorph was authorized in 2007 [3]. Remarkably, fungicide pressure has been intense in the north, where summers are moderate and precipitations are abundant. *P. viticola* resistance to CAAs has been reported in several countries in the world [4,5,6], and a high frequency has been found in some regions in Spain during the last few years (Summary of Annual Sensitivity Monitoring, www.frac.info, accessed on 7 October 2024). The CAA fungicides (FRAC group 40) specifically target oomycetes of the *Peronosporales* order, inhibiting the cellulose synthesis necessary in the formation of the cell wall. Two different single-point mutations in the cellulose synthase 3 gene (*CesA3)* of *P. viticola* can confer resistance to all active ingredients of the CAA group: G1105S and G1105V [7,8]. G1105V is detected worldwide at a low frequency and at a small number of sites [9]; however, in some European countries, the valine mutation may contribute to resistance [8,10,11]. Since *P. viticola* is a diploid oomycete, Blum et al. (2010) [7] studied and observed that CAA resistance is inherited in a recessive manner.

Fungicide resistance can be assessed with biological and molecular methods. Bioassays can be performed with basic economic equipment and for all classes of fungicides, but they are time consuming as well as labor intensive, and a large number of repetitions are required. Molecular assays are rapid and can detect low frequency alleles; however, they can only be applied when the resistance mechanism of the pathogen is well known. PCR-RFLP methods [12], allele-specific PCR methods [13], and LAMP assays [14] have been used for the rapid detection of fungicide resistance in different pathogens, but they are not quantitative.

Proper anti-resistance strategies require quantitative information (e.g., percentage of resistant over sensitive individuals) [1], so quantitative methods that can detect the frequencies of the mutant alleles in population samples need to be developed. In this sense, real-time qPCR assays have been developed for the quantification of the resistant allele frequency [15,16], but a reference standard curve is needed, which is often time consuming. ddPCR differs from classical PCR in that samples are partitioned to the level of single molecules and then amplified, and an all-or-none signal is obtained from each reaction. Subsequently, either the nature of the target molecule is analyzed or the number of target molecules is calculated [17]. In ddPCR, this partition and distribution of target DNA molecules occurs in multiple water-in-oil droplets. This technique, published by Hindson et al. (2011), enables the absolute quantification of target DNA in highly diluted target samples. By using fluorescent probes, reactions containing one or more target DNAs are classified as positive, while those reactions not containing target DNA are classified as negative. The number of target DNA molecules present is calculated using Poisson statistics [18].

In recent years, many different applications for ddPCR have been established and published, most of them in the medical field: blood pathogen detection in patients with suspected bloodstream infections [19], early detection of SARS-CoV-2 infection [20], or the detection of somatic mutations in B-cell and follicular lymphoma [21]. However, there are not many studies that use ddPCR in the agricultural biotechnology field, and there are even fewer in fungicide resistance monitoring. Miles et al. (2021) [22] developed a ddPCR assay for the quantification of QoI-sensitive and resistant isolates in *Erysiphe necator* mixed samples; Mavridis et al. (2021) [23] set up a ddPCR protocol for pesticide resistance monitoring in *Tetranychus urticae*, and Battistini et al. (2022) [24] established a ddPCR technique for the determination of the QoI resistance frequencies in *Zymoseptoria tritici.*

To generate recommendations for fungicide resistance management, it is essential to know whether the frequency of resistant individuals is low among the population, resistance is well established, or the population almost entirely consists of resistant individuals. Thus, it is critical to have monitoring techniques that can quantify this information. ddPCR is a highly reproducible technique for precise copy number quantification and target detection at very low concentrations. The objective of this study was to develop a ddPCR protocol for detecting and quantifying the frequency of G1105S and G1105V substitutions, which confer CAA fungicide resistance to *Plasmopara viticola*.

## 2. Materials and Methods

### 2.1. Field Sample Collection

Four commercial vineyards with cultivars sensitive to GDM were sampled during the 2022–2023 growing seasons in the Basque Country (northern Spain). A total of 40–50 leaves showing typical downy mildew symptoms (yellow oil spots on the adaxial surface with white downy covering on the abaxial surface) were randomly collected in each vineyard and considered to be representative of the *P. viticola* population for that vineyard. Collected leaves were kept in plastic bags and carried to the laboratory to be washed under running tap water. Leaves were kept overnight in the dark to obtain sporulation; after that, sporangia were collected in sterile distilled water and centrifuged at 13,000 rpm for 5 min to obtain a pellet. The pellet was stored at −20 °C until DNA extraction. Populations A, B, C, and D coming from the vineyards detailed in Table 1 were used as test samples to determine allele frequencies.

### 2.2. P. viticola Strains Isolation

Previous research demonstrated that it is rare for a single oil spot to contain more than one multilocus genotype [25]; for this reason, some single oil spots were excised from the collected leaf samples and considered single strains. Among these, three single oil-spot isolates—STR1, STR2 and STR3—carrying S1105S, V1105, and G1105 mutations, respectively, were employed as reference samples to optimize ddPCR conditions and assess the sensitivity and specificity of the protocol.

### 2.3. DNA Extraction

For reference samples, DNA was extracted from a 1 ${cm}^{2}$ sporulated leaf cutting, including leaf tissue and sporulation. For population samples, the DNA was extracted from the pellet of sporangia. In both cases, samples were disrupted using 3 mm tungsten carbide beads (Qiagen, Hilden, Germany) in the TissueLyser II (Qiagen, Hilden, Germany), after which DNA was extracted using the innuPREP Plant DNA Kit (Analytic Jena, Jena, Germany), according to the manufacturer’s instructions. All DNAs were tested for quality and concentration using a NanoQuant infinite M200PRO spectrophotometer (Tecan Trading AG, Männedorf, Switzerland).

### 2.4. Sequencing

The three reference samples used in this study were analyzed for the presence of the mutations S1105, V1105, and G1105. The *PvCesA3* gene fragment including the region codifying the mutations was amplified using primers described by Aoki et al. (2011) [26]. The PCR reactions were performed in a final reaction volume of 50 μL using 0.2 μM of each primer, 0.1 mM of each dNTP, 5 μL of 10× reaction buffer, 0.5 U of Taq Polymerase (Ibian Technologies, Zaragoza, Spain) and 80 ng of extracted DNA. After an initial incubation at 95 °C for 5 min, amplification was performed for 35 cycles with the following program: 95 °C for 20 s; 60 °C for 20 s; 72 °C for 20 sec. The final extension was carried out at 72 °C for 5 min.

**Figure 1:**
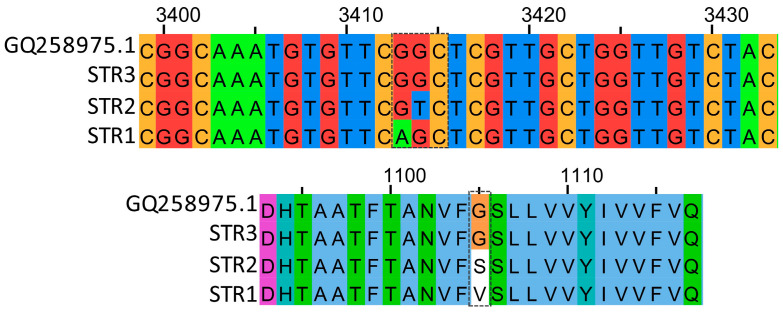
Alignment of GQ258975.1, STR1, STR2 and STR3 isolates for *CesA3* partial sequences.

Fragments consisting of 144 bp were purified using Nucleospin^®^ Gel and a PCR Clean-up Mini Kit (Macherey-Nagel, Düren, Germany) and then sequenced by Sanger sequencing using the forward primer employed for PCR amplification. Sanger sequencing was performed by Eurofins Genomics (Cologne, Germany), and each sample was sequenced two times. To investigate the point mutations in all strains, the nucleotide sequences were aligned using Jalview [27] (Figure 1) against the *PvCesA3* gene sequence of a reference *P. viticola* sequence (accession number GQ258975.1).

### 2.5. Equipment and ddPCR Reaction Setup

ddPCR experiments were carried out with a Rare Mutation Detection Assay, using the QX200 Drolet Digital PCR system (Bio-Rad Laboratories, Hercules, CA, USA). A pair of primers was designed to amplify a 65 bp long DNA fragment, and three probes (Table 2) were designed to target nucleotide positions 3413–3414 of the *CesA3* sequence from *P. viticola*, which was based on the GQ258975.1 reference sequence. Each probe was provided by the manufacturer (Bio-Rad) in a single tube, including both common primers (450 nM) and the dyed probe (250 nM).

Two duplex Rare Mutation Detection Assays were designed and optimized, each one detecting two target mutations: (i) PvCesA3_S assay (S1105 probe + G1105 probe) and (ii) PvCesA3_V assay (V1105 probe + G1105 probe). Each amplification reaction was prepared in a final volume of 22 µL to ensure that 20 µL of the mixture was then transferred to the DG8 cartridge (Bio-Rad). The following final concentrations of each component were utilized in all reactions: 1× Bio-Rad ddPCR™ supermix for probes (no dUTP, 2× concentrated), 1× each ddPCR assay primers—probe mix (20× concentrated, ready-to-use), double-distilled water and a variable amount of target DNA that ranged from 5 to 100 ng. Water-in-oil droplets were generated by a QX200 Droplets Generator (Bio-Rad) in DG8 cartridges (Bio-Rad) and amplified in a C100 Touch Thermal Cycler (Bio-Rad). Thermocycling conditions consisted of an initial denaturalization step of 10 min at 95 °C, which was followed by 44 cycles of 1:00 min at 94 °C (ramp rate of 2 °C/s) and 1 min at temperatures ranging from 55 to 60 °C (ramp rate of 2 °C/s) plus a final incubation time of 10 min at 98 °C. Droplets were left to rest for at least 30 min at 4 °C, after which absolute quantification was performed in the QX200 droplet reader (Bio-Rad). The generated raw data were analyzed by the QX Manager Software Standard Edition (v.2.1) to assign positive and negative droplets and to provide an absolute quantification of target DNA molecules as target copies/µL by Poisson statistics.

### 2.6. Optimization of ddPCR Conditions

Following the considerations of Digital MIQE Guidelines [28,29] for designing ddPCR experiments, the two assays (PvCesA3_S and PvCesA3_V) were optimized using the following means: varying the annealing temperature, which was used to optimize the distinction between positive and negative droplets; and varying the DNA template quantity, since the optimal template amount that results in a non-saturated population of positive droplets allows a reliable quantification. For each assay, an experiment was run at annealing temperatures of 55 °C, 58 °C and 60 °C. A second experiment was carried out at the optimum temperature using 5 ng, 25 ng and 100 ng of DNA template.

### 2.7. Sensitivity and Accuracy of the Probes

The sensitivity and accuracy of the assays were evaluated by their capability to discriminate between the alleles in mixed samples in vitro. For this purpose, equal concentrations (5 ng/µL) of DNA from resistant and sensitive isolates were mixed using the ratios reported in Table 3. Statistical analysis was conducted using RStudio v.4.2.2, where the linear correlation between copy numbers measured in ddPCR and corresponding percentages of the mixes were assessed.

### 2.8. Specificity and False-Positive Rate Estimation

When measuring rare genetic variants in samples containing predominantly wild-type mutations, it is important to assess specificity according to the Digital MIQE guidelines [29]. All three STR1, STR2 and STR3 isolates were separately amplified using both PvCesA3_S and PvCesA2_V assays to check cross-reactivity in the 2D plot.

For the estimation of the false-positive rate, wild-type DNA template was amplified in six independent wells, and an average of false-positive events per well was then estimated, as recommended in Bio-Rad Rare Mutation Detection Best Practices Guides.

### 2.9. Multiplexing

Multiplexing in ddPCR refers to multiple target sequence detection and quantification. In probe-based ddPCR, probes can only be labeled with two different fluorescent dyes (FAM and VIC/HEX), so multiplexing can be achieved by generating fluorescence signals of varying amplitude using the difference in probe concentration and amplification efficiency (Hou et al., 2023) [30]. Therefore, multiplex assays were carried out combining all three probes in the same PCR reaction mix. The same probes and reagents described in Section 2.5 were used. Final concentrations of each component in all multiplex reactions were as follows: 1× Bio-Rad ddPCR™ supermix for probes (no dUTP, 2× concentrated), 1× G1105 ddPCR assay primers–probe mix (20× concentrated, ready-to-use), 1× V1105 ddPCR assay primers–probe mix (20× concentrated, ready-to-use), 0.5× S1105 ddPCR assay primers–probe mix (20× concentrated, ready-to-use), double-distilled water and 5 ng of target DNA. All multiplex reactions were carried out in the selected optimum ddPCR conditions.

### 2.10. Determination of Allele Frequencies in Field Samples

Four field population samples were analyzed by the three ddPCR assays (two duplex and a multiplex), each one in triplicate. Reaction mixes without the DNA template were used as negative controls. Fractional abundances of the mutant alleles were calculated with the formula $\frac{S1105\;copy\;n{}^{\circ}+V1105\;copy\;n{}^{\circ}\;}{S1105\;copy\;n{}^{\circ}+V1105\;copy\;n{}^{\circ}+G1105\;copy\;n{}^{\circ}}$ on the basis of the fractional abundance formula of the Bio-Rad ddPCR applications guide [31]. In order to express the precision of the analysis, which is a measure of the closeness of agreement between replicate measurements [32], standard deviation values of the fractional abundance of each population were calculated.

## 3. Results

### 3.1. Optimization of ddPCR Conditions

**Figure 2:**
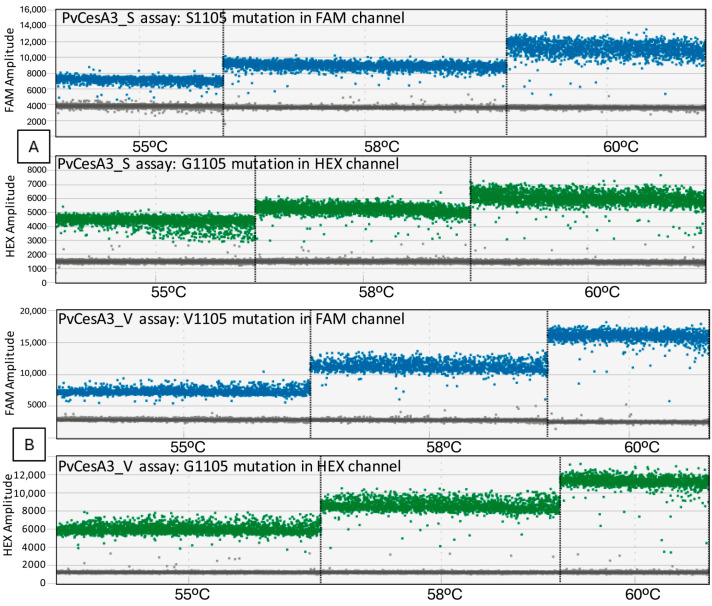
Amplitudes from FAM channel (blue) and HEX channel (green) obtained during the experiment to optimize the annealing temperature in a 1D chart generated by the QX Manager Software. (**A**) corresponds to the PvCesA3_S assay and (**B**) to the PvCesA3_V assay.

The clearest separation of positive and negative droplets occurred at 55 °C in both FAM and HEX channels in PvCesA3_S (Figure 2A) and in PvCesA3_V assay (Figure 2B).

High DNA quantities, such 100 ng, did not cause the channels to become saturated in any of the assays; however, 5 or 25 ng appeared to be the best amount of DNA, since separation between double-positive and wild-type clusters was more pronounced (data not shown). No positive droplets were detected in any of the NTCs, and the total event number ranged from 10,201 to 16,343 in PvCesA3_S assay and from 13,130 to 18,019 in PvCesA3_V assay.

### 3.2. Sensitivity and Accuracy of the Probes

**Figure 3:**
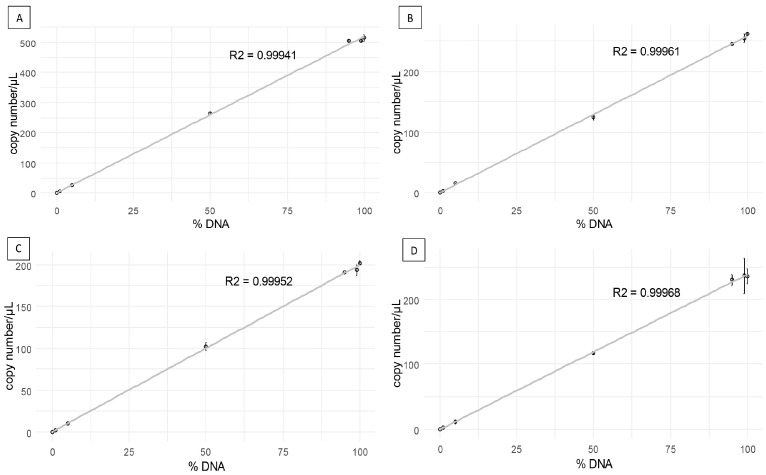
Linear correlation between values of measured copy number quantified by ddPCR (*Y*-axis) and the percentage of DNA from the DNA mixtures prepared with the STR1, STR2 and STR3 isolates. Each point represents the average of duplicates. (**A**) S1105 mutation copy number per µL in PvCesA3_S assay, (**B**) G1105 mutation copy number per µL in PvCesA3_S assay, (**C**) V1105 mutation copy number per µL in PvCesA3_V assay and (**D**) G1105 mutation copy number per µL in PvCesA3_V assay.

Since reference samples consisted of both leaf DNA and *P. viticola* DNA, it was not possible to previously determine the precise copy number in the mixes; however, all ddPCR measurements were proportional to the percentage of DNA in the generated DNA mixes (Figure 3). This is, both HEX and FAM fluorescent dyes measured properly the copy numbers in mixtures where the resistant allele was predominant or where the sensitive allele was predominant. The lowest frequencies that ddPCR were able to detect in this experiment were 0.38%, 1.79%, 1.14%, and 1.09%, corresponding to the 1% proportions of mutations S1105 and G1105 in PvCesA3_S assay as well as mutations V1105 and G1105 in PvCesA3_V assay, respectively. In the samples containing 100% of the reference DNA, neither allele copies of the other mutations nor any copies in the NTCs were detected.

### 3.3. Specificity and False-Positive Rate Estimation

**Figure 4:**
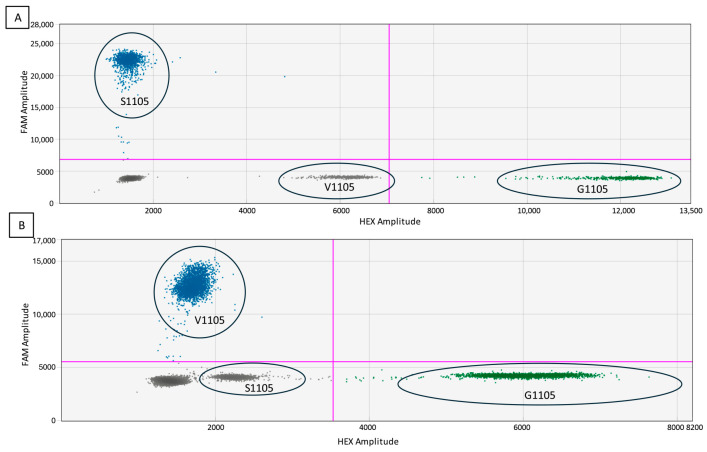
Two-dimensional (2D) cluster plot of droplet fluorescence for a Rare Event Detection (RED) assay. (**A**) PvCesA3_S assay and (**B**) PvCesA3_V assay. The pink lines represent the manually set threshold.

When testing specificity, a low-amplitude cluster was found in the HEX channel. In the case of PvCesA3_S assay (Figure 4A), the low amplitude cluster was caused by the HEX probe attaching to the V1105 mutation. In the case of PvCesA3_V assay (Figure 4B), the low amplitude cluster was caused by the HEX probe attaching to S1105 mutation. In either case, the clusters were clearly separated, so it was acceptable to threshold these clusters as negative for the mutant of interest, as shown in Figure 4. Nevertheless, to avoid thresholding problems in complex population samples from field, a multiplex assay was developed.

**Figure 5:**
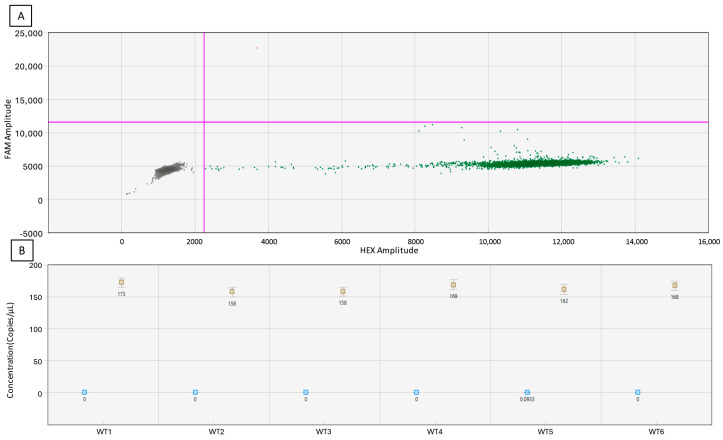
Estimation of false positive rate, where (**A**) 6 well merged 2D chart; (**B**) concentrations and error bars associated of independent wells. The pink lines represent the manually set threshold.

In the experiment below (Figure 5), false positive events were measured using the same WT DNA sample in six independent wells. In these individual wells, only one well had one FAM false positive droplet, and five wells had 0 FAM false positive droplets, giving an average of 0.17 false positive droplets per well.

### 3.4. Multiplexing

**Figure 6:**
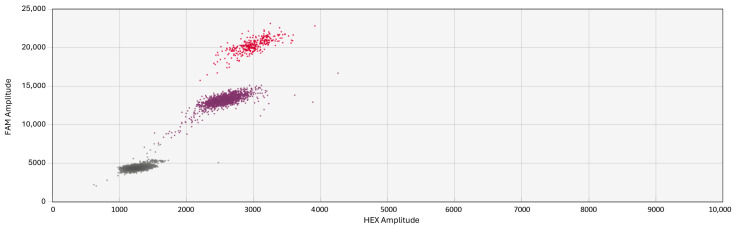
Two-dimensional (2D) chart of population sample B analyzed by multiplex assay. The higher cluster (red) corresponds to the mutation V1105 (amplitude: 15,000–22,000), whereas the lower cluster (purple) corresponds to the S1105 mutation (amplitude: 10,000–15,000).

Both mutant targets are visible in the FAM channel clearly separated at various amplitudes, as seen in Figure 6. Each has been assigned a distinct color with the software so that each one could be independently quantified. This multiplex assay resolved the specificity issue mentioned before where the WT probe was attached to the “free” S1105 or V1105 target and a cluster appeared in the HEX channel (Figure 4).

### 3.5. Determination of Allele Frequencies in Field Samples

The mean number of copies of each target mutation measured in the assays is detailed in Table 4. The mean fractional abundances and their standard deviations are also detailed, which revealed different mutation frequencies. The lowest frequency was found in population A, where chemical fungicides have never been applied. The highest frequencies were found in populations B and C, which were vineyards located in a high GDM pressure region. Population D had an intermediate resistance frequency, since this population came from a region with lower GDM pressure, which also means lower fungicide pressure.

## 4. Discussion

To our knowledge, this study presents the first development of a ddPCR protocol for quantifying the allele frequencies of S1105 and V1105 in *P. viticola* bulk samples collected from the field.

In ddPCR, there is no calibration curve needed for quantification, and the DNA present is quantified directly. This makes the quantification more reliable than real-time PCR, as real samples can have different amplification efficiencies than those obtained in setting the calibration curve [33].

Furthermore, ddPCR protocols are typically faster to establish than other molecular quantitative methods due to standardized reagents and sample processing. This standardization minimizes reproducibility issues both within and across laboratories. Additionally, ddPCR is more sensitive than qPCR, particularly in samples containing inhibitors, as the sample partitioning in ddPCR reduces the impact of inhibitors on quantification [34].

However, ddPCR tends to be more costly than qPCR due to the need for specific consumables such as gaskets, cartridges, and droplet oil for sample testing. According to Maheswari et al. (2021) [35], ddPCR was found to be 2.3 times more expensive than qPCR, while Van Heetvelde et al. (2017) reported that it was six times more expensive [36].

The assay itself also takes approximately two to three times longer compared to qPCR [18,35,37].

ddPCR relies on well-designed and optimized assays [28], which require preliminary research involving several steps for protocol optimization, which is a potentially labor-intensive process. According to the Bio-Rad Droplet Digital Applications Guide [31], at least 10,000 droplet generation events are needed for accurate data processing. In all the experiments described above, a total of 10,201 to 19,288 events were obtained. Higher event counts could be achieved, as suggested by Rowlands et al. (2019) [38], by incubating ddPCR plates on the cycler at 12 °C for at least 4 h after cycling and before transfer to the droplet reader, which significantly increases the total event number.

Two experiments evaluating a range of temperatures and DNA quantities were conducted to find the optimal PCR conditions for the clear separation of positive and negative droplets, optimizing both the thermal protocol and the amount of DNA to be utilized in the reaction. This was sufficient to obtain amplification conditions that were completely satisfactory. The tested temperatures were chosen based on the ones that performed the best in previous studies [24,39,40] and, similarly, the optimal annealing temperature was found to be 55 °C. None of the DNA quantities used in the experiments resulted in saturation, so 25 ng was chosen. For population samples, however, this amount was reduced to 5 ng because reference samples used in the experiment included a mixture of *P. viticola* and leaf DNA, and 5 ng was found to be a better amount for samples with pure *P. viticola* DNA coming from the field (data not shown).

With the purpose of detecting G1105S substitution in *Plasmopara viticola,* Aoki et al. (2011) [26] and Nanni et al. (2016) [6] have developed PCR-RFLP methods. An ARMS PCR has been developed by Zhang et al. (2017) [9] and a TaqMan-MGB Real-Time PCR by Huang et al. (2020) [41]. Nonetheless, as these methods are not quantitative, obtaining consistent data requires sampling and analyzing separately a very large number of isolates in each vineyard, because studying population samples would only reveal if the population is susceptible, mixed, or resistant. Furthermore, none of the existing research has addressed the G1105V substitution, as it is uncommon in most wine-growing regions. Sierotzki et al. (2011) [8] developed an allele-specific real-time qPCR technique for quantifying both G1105S and G1105V substitutions in bulk samples; however, this method can produce false positives and requires a standard curve.

ddPCR Rare Mutation Detection assays are designed to detect a sequence variant that is present at a very low frequency in a pool of wild-type backgrounds. With this method, deletions, insertions, and SNP variants can be targeted and precisely quantified. In our study, three ddPCR assays were validated to detect S1105 and V1105 mutations at low concentrations. Additionally, we conducted a test using artificially prepared mixes with varying mutation ratios to evaluate the technique’s capability for measuring mutations at higher frequencies. This second test demonstrated that both the PvCesA3_S and PvCesA3_V assays could consistently quantify mutation frequencies across populations with different resistance levels.

The ddPCR assays revealed different allele frequencies in the population samples analyzed, where population B showed a very high frequency of resistant alleles (100%, Table 4) and population A showed a very low frequency (7.42%, Table 4). These results align with the origin of the samples (Table 1). All A, B and C samples came from a territory where *P. viticola* pressure is very high and many chemical fungicide treatments are given each growing season, so high resistance was expected. In the case of population D, the sample came from a territory where GDM infections occur sporadically and lower resistance was expected. Predictably, population B, where chemical fungicides have never been applied, showed the lowest frequency. Interestingly, in population C, where no CAA fungicides were used during the last two seasons, a small number of sensitive alleles (0.77%) was found compared to the B population, where CAA fungicides were applied every season. Measurements in the ddPCR showed fractional abundance standard deviations ranging from 0.04 to 1.74 (Table 4). Both duplex and multiplex techniques were shown to reliably determine the fractional abundances in all the samples. However, overall, the multiplex exhibited a smaller standard deviation due to three mutations that could be read and measured in a single well (two wells are required in the duplex), and operator manipulation errors are less reflected in the measurements. Additionally, the multiplex improves the method’s diagnostic potential by saving time, effort, and expensive reagents [30].

Due to the diploid nature of *P. viticola*, mutations in the coding sequence may not always result in mutant phenotypes; in fact, S1105 and V1105 mutations are recessive in *P. viticola* [7]. This must be considered when extrapolating ddPCR results, because an intermediate–high S1105 and V1105 frequency does not necessarily imply resistance, and bioassays may be required to determine ${EC}_{50}$ values.

This study presents a quantitative and highly sensitive approach that improves upon currently available methodologies for monitoring *Plasmopara viticola*. While considerations regarding the time required, technical expertise for droplet generation across numerous samples, and associated costs are important, our results indicate that this ddPCR protocol is suitable for large-scale monitoring studies. This tool can facilitate the early detection and quantification of the initial stages of resistance evolution, enabling the observation of shifts in allelic composition within populations over time.

## 5. Conclusions

The ddPCR protocol outlined in this study has demonstrated high accuracy, precision, sensitivity, and reproducibility in quantifying S1105 and V1105 mutations within bulk samples of *Plasmopara viticola*. Utilizing this quantitative molecular data alongside biological assays is fundamental for formulating evidence-based recommendations in fungicide resistance management. This approach enables the adaptation of management strategies across a spectrum of resistance scenarios from susceptible to highly resistant populations. Furthermore, the versatility of this methodology suggests that similar ddPCR assays could be developed to detect mutations associated with fungicide resistance across various fungicide classes and plant pathogens, potentially broadening its applicability within agricultural pathogen management. Utilizing ddPCR in resistance monitoring programs may deepen our understanding of resistance dynamics at the population level, supporting the development of more sustainable and effective fungicide application strategies.

## Figures and Tables

**Table 1:** Origin and information of four *P. viticola* field samples.

Population	Village	PDO ^1^	Cultivar	Fungicide Applications in 2023
A	Olaberria	Getariko Txakolina	Hondarrabi Zuri	Never exposed to CAA fungicides
B	Muxika	Bizkaiko Txakolina	Hondarrabi Zuri	Ampexio (mandipropamid + zoxamide) Java (valifenalate + folpet)
C	Izurtza	Bizkaiko Txakolina	Hondarrabi Zuri	No CAA fungicide applications
D	Buradon Gatzaga	Rioja	Tempranillo	No CAA fungicide applications

^1^ PDO = Protected Designation of Origin.

**Table 2:** Primer and probe sequences developed for ddPCR assays.

Name	Sequence (5′ to 3′)	Probe Dye
Forward primer	ACGGCTGCTACCTTTAC	
Reverse primer	ACAAACACGACAATGTAGAC	
G1105 probe	AATGTGTTCGGCTCGTT	HEX
V1105 probe	TTCGTCTCGTTGCTGG	FAM
S1105 probe	AATGTGTTCAGCTCGTTG	FAM

**Table 3:** Composition of mixed DNA samples. For the PvCesA3_S assays, isolates STR1 and STR3 were mixed to obtain 7 mixes. For the PvCesA3_V assays, STR2 and STR3 were mixed to obtain 7 mixes.

		Mix 1	Mix 2	Mix 3	Mix 4	Mix 5	Mix 6	Mix 7
PvCesA3_S	S1105	0%	1%	5%	50%	95%	99%	100%
	G1105	100%	99%	95%	50%	5%	1%	0%
PvCesA3_V	V1105	0%	1%	5%	50%	95%	99%	100%
	G1105	100%	99%	95%	50%	5%	1%	0%

**Table 4:** Fractional abundances of mutations in *P. viticola* populations. S1105, V1105 and G1105 correspond to the three replicates mean copies/µL measured in the ddPCR assays.

Populations	Duplex					Multiplex				
	S1105	V1105	G1105	Fractional Abundance (%)	SD ^1^	S1105	V1105	G1105	Fractional Abundance (%)	SD ^1^
A	35.30	3.55	602.84	6.62	0.36	23.85	2.18	325.00	7.42	0.04
B	424.5	73.83	0.00	100	0.00	354.17	66.50	0.00	100	0.00
C	280.67	87.77	3.79	98.98	1.42	261.67	88.77	2.70	99.23	0.20
D	144.7	37.03	116.15	61.00	0.66	216.67	64.00	201.17	59.16	1.74

^1^ SD = Standard deviation.

## Data Availability

The original contributions presented in the study are included in the article; further inquiries can be directed to the corresponding author.
